# Deciphering the phenotypic spectrum associated with *MIA3*-related odontochondrodysplasia

**DOI:** 10.1038/s10038-025-01328-y

**Published:** 2025-03-21

**Authors:** Mohamed S. Abdel-Hamid, Rasha M. Elhossini, Sherif F. Abdel-Ghafar, Mennat Mehrez, Mona S. Aglan, Nehal F. Hassib

**Affiliations:** 1 Medical Molecular Genetics Department, Human Genetics & Genome Research Institute, National Research Centre, Cairo, Egypt; 2 Clinical Genetics Department, Human Genetics & Genome Research Institute, National Research Centre, Cairo, Egypt; 3 Orodental Genetics Department, Human Genetics & Genome Research Institute, National Research Centre, Cairo, Egypt; 4 Dental Consultant, School of dentistry, New Giza University, Giza, Egypt

**Keywords:** Calcium and phosphate metabolic disorders, Multihormonal system disorders

## Abstract

Odontochondrodysplasia (ODCD) is a rare skeletal dysplasia characterized by short stature, skeletal deformities, and dentinogenesis imperfecta (DI). Although the majority of cases were associated with biallelic variants in *TRIP11*, one study described a homozygous truncating variant in *MIA3*, encoding TANGO1, in four sibs with ODCD in association with insulin-dependent diabetes, hearing loss, obesity, and intellectual disability. Subsequently, a homozygous truncating variant in the luminal domain of TANGO1 was identified in a fetus with a lethal skeletal dysplasia and fetal hydrops. Herein, we describe two unrelated patients with a distinct phenotype including severe short limbs, short stature, metaphyseal dysplasia, dysmorphic facies, lax joints, and DI. Other variable features were scoliosis, squint, and cardiac problems. Exome sequencing revealed two homozygous *MIA3* variants in the luminal domain of TANGO1, c.354+2T>G and p.Cys38Phe. The c.354+2T>G variant was confirmed by investigating the patient’s mRNA to result in exon 3 skipping and an inframe deletion of 29 amino acids. Our patients lacked the extra-skeletal manifestations noted in the four sibs with *MIA3* variant. However, they had more severe skeletal deformities closely resembling those observed in patients with *TRIP11* variants. Our study suggests the presence of a phenotypic spectrum associated with *MIA3* variants including ODCD with milder skeletal deformities, a classic ODCD with severe skeletal deformities, and a lethal skeletal dysplasia at the severe end of the spectrum. Although the striking phenotypic variability appears to be related to the type and or the location of the *MIA3* variants, the influence of other factors cannot be ruled out.

## Introduction

Spondylometaphyseal dysplasia (SMD) comprises a heterogenous group of skeletal dysplasias marked by characteristic radiological features such as flattened vertebrae and varying metaphyseal irregularities. The clinical diagnosis of the different SMD subtypes is very challenging due to the overlapping manifestations and often requires advanced genomic testing for accurate diagnosis and counseling [1, 2]. Odontochondrodysplasia (ODCD), also known as Goldblatt syndrome, is a very rare form of SMD characterized by mesomelic shortening of the tubular bones, scoliosis, ligamentous laxity, cone-shaped epiphyses, and dentinogenesis imperfecta (DI). ODCD was initially attributed to biallelic variants in *TRIP11* (OMIM: 604505) which encodes a protein crucial for the structural maintenance of the Golgi apparatus. To date, 16 patients from 12 unrelated families with *TRIP11* variants have been described in the literature [3–6].

A homozygous variant in the *MIA3* (OMIM: 613455), encoding the TANGO1 protein, has been identified in four sibs with ODCD alongside other systemic manifestations like insulin-dependent diabetes, hearing loss, obesity, and mild intellectual disability [7, 8]. TANGO1 (Transport and Golgi Organization 1) plays a vital role in collagen secretion by facilitating the export of large cargoes—such as collagens I, II, III, IV, and IX—from the endoplasmic reticulum (ER) to the Golgi apparatus. Collagen export is critical for bone mineralization, connective tissue strength, and dental development [8].

In this study, we describe the clinical, radiological, and orodental findings of two new families with ODCD harboring novel homozygous *MIA3* variants. We compare their data with the only published family to refine and deep phenotyping this extremely rare form of ODCD.

## Materials and methods

### Patients

The study included two unrelated patients recruited from the Limb Malformations and Skeletal Dysplasia Clinic (LMSDC), Centre of Excellence, at the National Research Centre (NRC), Cairo, Egypt. The two patients were subjected to meticulous medical history taking, three-generation pedigree construction, extensive clinical and radiological examinations (skeletal and orodental), and anthropometric measurements including weight, height, and head circumference. Other investigations included abdominal ultrasound, CT brain, echocardiography, and fundus examination. In addition, laboratory analysis of basal growth hormone, growth hormone with stimulation, thyroid function tests, serum calcium, phosphorus, alkaline phosphatase, parathormone hormone, blood sugar level, glycosylated hemoglobin, immunological profiles, and renal function tests were performed.

### Methods

The study was approved by the Scientific Committee of the Orodental Genetics Department and the Medical Research Ethics Committee of NRC in accordance with the Declaration of Helsinki (Approval number: 20068). Genomic DNA was extracted from the patients and available family members using a standard extraction procedure after having signed informed consents from the parents. Whole exome sequencing was performed for the two patients using the SureSelect Human All Exome 50 Mb Kit (Agilent, Santa Clara, CA, USA) and analyzed on Illumina NovaSeq 6000 (Illumina, San Diego, CA, USA). The obtained sequences were aligned to UCSC human genome GRCh37/hg19 and variants were verified using the GATK pipeline. Annotation of variants was performed using BaseSpace Variant Interpreter Server. Segregation analysis in the parents and healthy siblings was carried out using Sanger sequencing. To study the effect of the new *MIA3* splice variant, total RNA was extracted from Patient 1 leukocytes followed by cDNA synthesis and sequencing (Supplementary Methods).

## Results

The detailed clinical, radiological, oro-dental, and genetic findings of the two patients are summarized in Table 1.

**Table 1:** The demographic, clinical, and genetic data of patients with biallelic *MIA3* variants

	This study			Lekszas et al. [8]			
	Family 1	Family 2		Family 1			
	Patient 1	Patient 2		Patient 1	Patient 2	Patient 3	Patient 4
Age	5 years	1 year 9 months		32 years	30 years	14 years	12 years
Sex	Male	Male		Male	Male	Male	Male
Origin	Egypt	Egypt		Turkey			
Parental consanguinity	+	+		+			
Short stature	Disproportionate short limbs, short stature			Proportionate short stature			
Growth retardation	+	+		**+**	**+**	**+**	**+**
Mild intellectual disability	−	−		**+**	**+**	**+**	**+**
Relative macrocephaly	+	+		NA			
Ophthalmological findings	−	+ Squint		**+** Mild retinopathy	**−**	**−**	**+** Mild retinopathy
**Craniofacial findings**							
Full cheeks	+	+		NA			
High nasal bridge	+	+		**+**	**+**	**+**	**+**
Low set ears	+	**+**		**−**	**−**	**−**	**−**
Short philtrum	+	**+**		**+**	**+**	**−**	**−**
Flat philtrum	+	**−**		NA			
Fissured lower lip	+	**+**					
Microstomia	+	−					
**Skeletal findings** (clinical & radiological)							
Flared ribs	+	+		NA			
Short chest	+	+					
Platyspondyly	+	**+**		**+**	**+**	**+**	**+**
Scoliosis	+ Mild	−		**−**	**−**	**+**	**+**
Kyphosis	+	+		**−**	**−**	**−**	**−**
Lordosis	+	+		**−**	**−**	**−**	**−**
Metaphyseal dysplasia	+	+		NA			
Prominent knees	+	+		**+**	**+**	**+**	**+**
Genu valgum	+	−		NA			
Small hands	+	+		**+**	**+**	**−**	**−**
Brachydactyly of fingers	+	+		**+**	**+**	**+**	**+**
Brachydactyly of toes	−	−		**+**	**+**	**−**	**−**
Clinodactyly of 5^th^ finger	+	−		**+**	**+**	**+**	**+**
Cone shaped epiphyses	−	−		**+**	**+**	**+**	**+**
Broad joints	+	+		NA			
Lax joints	+	+					
**Oro-dental findings**							
Dentinogenesis imperfecta	**+**	**+**		**+**	**+**	**+**	**+**
Mandibular Prognathism	**+**	**+**		**−**	**−**	**−**	**−**
Tongue thrust	**+**	**+**		**−**	**−**	**−**	**+**
Others	Prominent premaxilla, premature eruption, malocclusion, high arched palate, everted lower lip	Habitual open mouth, macroglossia, thin upper lip		**−**	**−**	**−**	**−**
**Other findings**							
Insulin dependent diabetes mellitus, obesity, hearing defect, sensorineural hearing loss	−	−		**+**	**+**	**+**	**+**
Heart abnormalities	−	+ trivial mitral regurge and tricuspid regurge		**−**	**−**	**−**	**−**
**Molecular findings**							
*MIA3* (NM_198551) Variant	c.354+2T>G (p.Val90_Asp118del)		c.113G>T (p.Cys38Phe)	c.3621A>G (p.Val1204SerfsTer10)			

*NA* Not available

### Patient 1

**Fig. 1:**
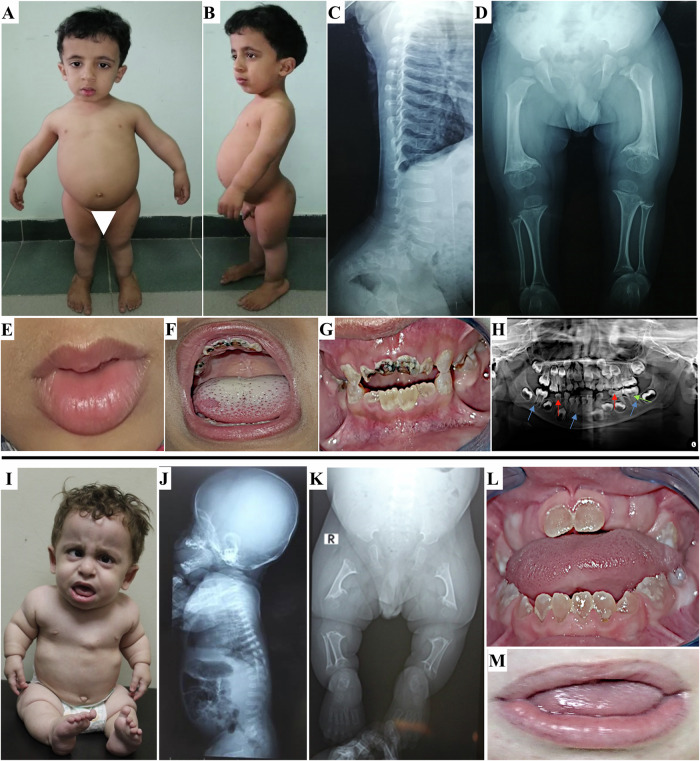
Patient 1 at the age of 5 years. **A**, **B** Frontal and lateral views showing relative macrocephaly, frontal and parietal bossing, high forehead, flat philtrum, low set ears, short neck, short narrow chest, depressed lower sternum, distended abdomen, exaggerated lumbar lordosis, broad joints, genu valgum and flat feet. **C**, **D** X-ray spine lateral view and lower limbs (AP view) showing mild platyspondyly, globular shaped vertebrae, flat acetabulum, the greater trochanter of the left femur was separated and posteriorly displaced, rounded femoral head, broad femoral neck, wide knee space, globular epiphysis around knees, both fibulae were proximally short, broad cupped frayed sclerotic metaphysis and short bowed long bones. **E** Microstomia and everted fissured lower lip. **F** Very high arched palate and geographic tongue. **G** Opalescent dentin and anterior open bite. **H** Panoramic radiograph showing bulbous crowns of deciduous teeth, needle like roots anterior lower teeth with obliterated pulp (blue arrow), thin roots of molars (blue arrows), wide pulp chamber (red arrow), pulp stone (green arrow), premature eruption of lower right first premolar, and very thin dentin layer. Patient 2 at the age of 1 year and 9 months. **I** Frontal view showing relative macrocephaly, high forehead, frontal and parietal bossing, squint, bulbous nose, simple ears, low set ears, short neck, short narrow chest, pigeon chest, flared ribs and skin creases. **J**, **K** Skeletal survey showing platyspondyly, globular shaped vertebrae, flat acetabulum, absent femoral head, dumbbell shaped short long bones, wide knee spaces, faint around knees epiphyseal ossification, broad cupped frayed metaphysis, and bone rarefaction. **L** Intraoral photo shows opalescent dentin of the erupted deciduous dentition. **M** Thin upper lip, fissured lower lip, open mouth and protruded tongue

A 5-year-old male patient, the second child to healthy consanguineous Egyptian parents. Gestational history was unremarkable and he was born full-term with an average birth weight of 2.7 kg (SDS −1.38). After birth, he was incubated for 23 days on CPAP due to respiratory distress. He had a history of chest intermediate care admission at the age of 7 months due to pneumonia. General examination at the age of 9 months demarcated wide open anterior and posterior fontanels (8 & 4 cm, respectively), relative macrocephaly, distinctive facies with down slanting of eyelids, deformed chest, distended abdomen, mild kyphosis, micromelia, lax joints, and hypotonia (Fig. 1A, B). Examination of external genitalia revealed normal male genitalia and bilateral hydrocele. Anthropometric measurements were: weight 5.5 kg (SDS −3.3), height 54 cm (SDS −7.13), and head circumference 44.2 cm (SDS −0.4). He had short limbs as the upper to lower segment ratio was 2.6. The patient had delayed motor milestones as he walked at age of 3 years. He had an average mentality, recurrent chest infections, abnormal gait and progressive skeletal deformities. At the last follow up, at the age of 5 years, his weight was 10.5 kg (SDS −3.7), height 71.2 cm (SDS −7.25), and his head circumference 51.3 cm (SDS + 1.5). Vineland scale revealed an IQ score of 92. Skeletal survey showed platyspondyly and severe metaphyseal dysplasia denoting spondylometaphyseal dysplasia in addition to short bowed long bones (Fig. 1C, D). Oro-dental examination revealed microstomia, everted fissured lower lip, high arched palate, and dentin defects with obvious opalescence of the appearing dentitions (Fig. 1E–G). Panoramic view showed thin enamel over bulbous crowns of deciduous teeth, wide pulp chambers, needle like roots of anterior teeth with obliterated pulp, thin roots of erupted molars, as well as a pulp stone in the upper right 1^st^ permanent molar and lower left 1^st^ permanent molar. Premature eruption of the lower right 1^st^ permanent premolar was also seen (Fig. 1H).

### Patient 2

A 1-year and 9-month- old male patient, the only child to healthy consanguineous Egyptian parents. He was born at full-term and his birth weight was 3 Kg (SDS −0.9). After birth, he was incubated for 7 days on CPAP due to respiratory distress. General examination revealed brachycephaly, relative macrocephaly, distinctive facies, squint, simple ears, low set ears, pigeon chest, distended abdomen, mild kyphosis, lordosis, micromelia, lax joints, and brachydactyly (Fig. 1I). External genitalia examination showed normal male genitalia. Neurological examination revealed generalized hypotonia and hyporeflexia. His anthropometric measurements were: weight 7 kg (SDS −4.14), height 61 cm (SDS −7.11), and head circumference 49 cm (SDS −0.57). He had short limbs as the upper to lower segment ratio was 2.4. Complete eye evaluation revealed bilateral squint with normal fundi. Echocardiography showed trivial mitral regurge and tricuspid regurge. Skeletal survey revealed shortening of long bones and spondylometaphyseal dysplastic changes in the form of platyspondyly and broad dysplastic metaphysis (Fig. 1J, K). Oro-dental examination showed thin upper lip, fissured lower lip, open mouth, protruded tongue, and dentin defects with obvious opalescence of the appearing dentitions (Fig. 1L, M). The patient was too young to provide any panoramic X-ray.

### Molecular results

**Fig. 2:**
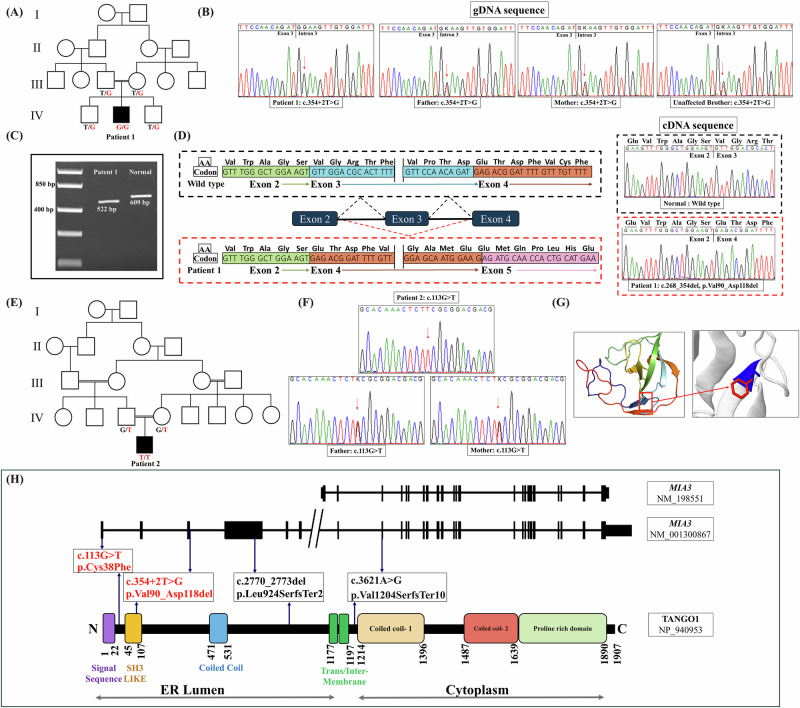
**A** Pedigree of Family 1. **B** Portions of the sequencing electropherograms (gDNA) showing the segregation of the *MIA3* variant identified in the family. The arrow indicates the site of variant. **C** A 2% agarose gel showing partial amplification of the cDNA of the *MIA3* (from exons 2–4) in Patient 1 and a normal control subject. **D** Schematic diagram showing exon 3 skipping and part of the sequencing electropherograms of the cDNA fragment of Patient 1 in comparison to normal control subject. **E** Pedigree of Family 2. **F** Portions of the sequencing electropherograms showing the identified *MIA3* missense variant in the patient and his parents. The arrow indicates the site of variants. **G** Structural analysis of the TANGO1 with the p.Cys38Phe variant using Missense3D, predicting a disruption of a disulfide bond and increased steric clashes, potentially impairing protein function. **H** Schematic diagram of the two long and short transcripts of *MIA3* and TANGO1 domains showing all reported variants and their locations. Variants identified in this study are in red

Exome sequencing identified biallelic variants in *MIA3* (NM_198551) as the likely cause of the patients’ phenotype. A homozygous variant in the donor splice site of exon 3 was identified in Patient 1, c.354+2T>G. The variant was found in the heterozygous state in the parents and the unaffected brothers (Fig. 2A, B). To assess the impact of the new splice variant on the protein, we partially amplified the *MIA3* (exons 2–4) using cDNA from the patient and a normal control individual. Agarose gel electrophoresis revealed a shorter band in the patient compared to the normal control (Fig. 2C). Subsequent sequence analysis confirmed that the splice variant induced skipping of exon 3 resulting in the removal of 29 amino acids from the protein, thereby leading to an in-frame deletion effect (p.Val90_Asp118del) (Fig. 2D). In Patient 2, a homozygous missense variant in exon 1 resulting from the substitution of a conserved cysteine by phenylaniline at position 38 (c.113G>T, p.Cys38Phe) was identified. The missense variant segregated nicely with the phenotype in the family as both parents were heterozygotes (Fig. 2E, F). The two variants were absent in gnomAD v.4 and our in-house database of over 2500 exomes of Egyptian origin and multiple in-silico prediction tools supported their pathogenicity (Supplementary Table 1). Furthermore, Missense3D predicted that the substitution of p.Cys38 would disrupt the three-dimensional structure of the protein (Fig. 2G). According to the ACMG classification of variants, the c.354+2T>G should be classified as a pathogenic variant (PVS1, PM2, PM4, PP3) and the c.113G>T (p.Cys38Phe) as a variant of uncertain significance (PM2, PP3).

## Discussion

TANGO1 plays an essential role in the export of large cargoes, such as collagen, from the ER to the Golgi apparatus, with two main isoforms: TANGO1L (long) and TANGO1S (short). TANGO1L includes a signal peptide, coiled-coil, proline-rich regions, and a critical SH3-like domain, which enables interaction with Sec23, an essential step in organizing ER exit sites (ERES) and recruiting Sec16 to initiate COP-II vesicle formation for transporting bulky cargoes. This SH3-like domain also allows TANGO1L to bind collagen indirectly through the chaperone HSP47, directing collagen to ERES for transport. Disruption of TANGO1L results in a fragmented Golgi, distended ER, retained ER cargo, and elevated ER stress markers, emphasizing its importance in cellular transport and collagen secretion [9, 10]. TANGO1S, on the other hand, is a spliced isoform sharing some cytoplasmic domains with TANGO1L but lacks the signal peptide, SH3 domain, and coiled-coil domain. Consequently, TANGO1S has a limited role in the secretory pathway, as it cannot directly interact with collagen. While it is expected that TANGO1-short binds the same cytoplasmic proteins as TANGO1-long, this has not yet been directly confirmed [11].

In this study, we describe two new patients harboring homozygous *MIA3* variants. The phenotype of our patients showed some overlapping features with the four sibs described by Lekszas et al. [8]. The six patients had short stature, generalized bone rarefaction, platyspondyly, brachydactyly, fifth-finger clinodactyly, dentinogenesis imperfecta, and recurrent chest infections. However, the characteristic cone-shaped epiphyses noted in the four Turkish sibs were absent in our patients. In addition, the extra-skeletal manifestations (hearing loss, retinopathy, hydronephrosis, microalbuminuria, diabetes, primary obesity, and intellectual disability) were missing in our patients [8]. In contrast, our patients displayed striking disproportionate short limb short stature and severe skeletal dysplasia and had additional new findings like distinctive facial dysmorphism, joint laxity, and cardiac problems. The absence of the previously reported extra-skeletal manifestations may be related to the progressive nature of the disease and the different ages at presentation between our patients and the reported patients.

It has been confirmed that TANGO1 directly influences the pro-collagen formation in the endoplasmic reticulum and maintains the development of normal dentin [11, 12]. Therefore, it was not surprising to find DI as a constant finding in previously reported patients with *MIA3* variants [8] and our two patients. Interestingly, Patient 1 had very thin roots (needle like) which was also observed in three of the four sibs described by Lekszas et al. [8] highlighting root anomalies as part of the dental phenotype associated with *MIA3* variants. In addition, Patient 1 exhibited early eruption of the lower right 1^st^ permanent premolar which might have partly occurred due to premature loss of their predecessors. This patient lost his teeth prematurely due to caries and not due to juvenile periodontitis as noted in two of the four patients described by Lekszas et al. [8]. The association of diabetes and periodontal problems was previously proved [13].

The phenotype of our patients closely resembles that of *TRIP11*-related ODCD (Table 2). Both *MIA3* (encoding TANGO1) and *TRIP11* (encoding GMAP210) play key roles in a shared pathway involved in the secretion of extracellular matrix (ECM) components (Supplementary Fig. 1). While TANGO1 facilitates the packaging of bulky cargo, particularly collagen, into COPII vesicles, GMAP210 is essential for maintaining Golgi structural integrity, organizing the trafficking routes, and enabling proper processing and transport of large ECM molecules. Additionally, *TRIP11* variants impair the differentiation of chondrocytes into hypertrophic chondrocytes, which are responsible for producing type X collagen (COL10A1) (Wehrle et al., [3]). Overall, the disruption of ECM secretion due to variants in either gene can result in an ODCD phenotype.

**Table 2:** The clinical features of previously reported patients with *TRIP11* variants and our two patients with *MIA3* variants

	Previously reported patients with *TRIP11* variants (n/16)	Our patients with *MIA3* variants (n/2)
Sex	6 males / 10 females	2 males
Dentenogenesis imperfecta	14/16 (2 patients died early)	2/2
Spondylometaphyseal dysplasia	16/16	2/2
Short stature	15/16	2/2
Relative macrocephaly	14/16	2/2
Small chest	13/16	2/2
Pectus carinatum	6/16	1/2
Scoliosis	8/16	1/2
Mesomelic shortening	14/16	0/2
Brachydactyly	15/16	2/2
Joint hyperextensibility	9/16	2/2
Extraskeletal manifestations	Pulmonary hypoplasia (4/16) Polycystic Kidneys (1/16) Nephronophthisis (1/16) Hydrocephalus (1/16)	Congenital heart defects (1/2) Squint (1/2)

So far, only one *MIA3* variant has been associated with ODCD [8]. This variant is a synonymous (c.3621A>G) that was confirmed to lead to skipping of exon 8 and early protein truncation (p.Val1204SerfsTer10) in most transcripts and impacting the initiation of the cytoplasmic portion of TANGO1 resulting in reduced collagen secretion. In this study, we identified two new variants extending the number of *MIA3* causative variants, c.354+2T>G and p.Cys38Phe. The c.354+2T>G variant identified in Patient 1 disrupts normal splicing, leading to exon 3 skipping and a subsequent 29-amino acids deletion in the TANGO1. This in-frame deletion may compromise the structural integrity of TANGO1 and its function in cargo export. In the second patient, the identified missense variant (p.Cys38Phe) affects a conserved cysteine residue which is critical for maintaining TANGO1’s three-dimensional conformation. In-silico tools and structural prediction software indicated that substituting cysteine with phenylalanine is expected to disrupt the protein’s stability, impairing its ability to facilitate ER export of collagen and other large cargo proteins.

*MIA3* variants have been associated with several non-skeletal features as noted in our study and the report of Lekszas et al. [8]. This can be related to the fact that TANGO1 regulates the secretion of many cargoes. For instance, TANGO1 has been implicated in mucin export in Drosophila salivary glands [14] which can explain the recurrent chest infections noted in the patients. Additionally, *MIA3* has been reported to be expressed in heart tissue, although not abundantly [15]. In this regard, a single-nucleotide polymorphism (c.3169+315G) has been associated with an increased risk to coronary artery disease; however, the pathological pathway connecting this variant to cardiac issues remains incompletely understood [16]. Unlike the patients of Lekszas et al. [8], our patients did not exhibit hearing loss, hydronephrosis, microalbuminuria, diabetes, primary obesity, or intellectual disability. This may be attributed to TANGO1 short isoform which remains intact and unaffected by the identified variants in our study.

Based on the phenotypes of previously reported patients with *MIA3* variants, we might speculate the presence of a spectrum of skeletal dysplasias with variable severity and presentation. At one end of this spectrum lies a form of ODCD characterized by milder skeletal deformities as seen in the four patients reported by Lekszas et al. [8] followed by an intermediate form with classic ODCD which was observed in our two patients. At the severe end of spectrum, there is a lethal phenotype characterized by almost absent bone formation, fetal hydrops, as well as presumed biliary atresia and hepatomegaly as noted in the fetus described by Guillemyn et al. [17]. This fetus was found to harbor a homozygous frameshift variant (c.2770_2773del, p.Leu924SerfsTer2) which affected the luminal portion of TANGO1 and mimicked a complete loss-of-function situation, similar to that observed in *MIA3*−/− pups, which displayed early neonatal lethality associated with osteochondrodysplasia, lack of bone mineralization, dwarfism, and defective collagen secretion [17].

TANGO1 does not appear to be haploinsufficient as several heterozygous loss-of-function-variant carriers are documented in large population databases as ExAC and gnomAD. However, a recent study identified a heterozygous frameshift variant (c.5637dup, p.Leu1880ThrfsTer6) in a 49-year-old woman and her 14-year-old daughter, both exhibited hypermobile joints resembling an Ehlers-Danlos syndrome phenotype [18]. In general, the joint laxity noted in our subjects can be explained by the clear relation between TANGO1 and collagen.

In view of phenotype-genotype correlations, the type and/ or location of *MIA3* variants are likely to influence the severity of the phenotype. Truncating variants appear to be associated with a lethal phenotype when located in the N-terminal region [17]. In contrast, patients with missense or in-frame deletion variants, allowing for the retention of the entire cytoplasmic domain, present with a classic ODCD phenotype when located in the N-terminal region. A non-lethal milder ODCD phenotype with systemic extra-skeletal manifestations may be seen when variants present at the beginning of the cytoplasmic portion of the protein [8] (Fig. 2H).

In conclusion, our study highlights the phenotypic spectrum associated with *MIA3* variants which includes ODCD with mild skeletal phenotype and additional extra-skeletal features, intermediate classic ODCD, and a lethal skeletal dysplasia mimicking knockout animal models. We propose the presence of genotype-phenotype correlations as the type and location of *MIA3* variants appear to significantly influence the disease severity and associated manifestations. The identification of more patients with different *MIA3* variants would help to confirm this observation. Although ODCD shares many overlapping features with other forms of SMDs, the presence of DI is pathognomonic and warrants genetic testing of *TRIP11* and *MIA3* to identify the causative variants and provide proper genetic counseling for the families.

## Supplementary information

Supplementary Methods

Supplementary Figure 1

Supplementary Table 1

Supplementary Figure 1 Legend
