# Supplementary material for: Deciphering the phenotypic spectrum associated with MIA3-related odontochondrodysplasia
Source: J Hum Genet. 2025 Mar 21;70(5):257–63. doi: 10.1038/s10038-025-01328-y (PMC11964919; doi:10.1038/s10038-025-01328-y)
Supplement: Supplementary file 1 — Supplementary Methods [file 10038_2025_1328_MOESM1_ESM.docx]

**Supplementary Methods**

**Segregation of the *MIA3* variants**

The two *MIA3* variants identified by exome sequence were confirmed in the parents and the unaffected brother of Patient 1 using Sanger sequencing. The regions encompassing the two variants (exons 1 and exon 3) were amplified using the following primers sequence:

Exon 1F: 5’- CCCCGAGGTGACCACAAC -3‘

Exo 1R: 5’- GAGAAGGCAGACACAGGGAC -3‘

Exon 3F: 5’- TGAGGTTTGCAGCTCTATCG -3‘

Exon 3R: 5’- AAGCAGAGCGTGCAGTGAG -3‘

Primers were designed by ExonPrimer software. PCR cycling conditions were: initial denaturation at 96°C for 5 min; 30 cycles of denaturation at 96°C for 30 sec; annealing at 62°C for 30 sec; extension at 72°C for 30 sec, and a final extension at 72°C for 5 min. PCR products were purified using Exo-SAP PCR Clean-up kit (Fermentas, Germany) and sequenced in both directions using the BigDye Terminator v3.1 Cycle Sequencing Kit (Applied Biosystems, Foster City, CA, USA) and analyzed on the ABI Prism 3500 Genetic Analyzer (Applied Biosystems) according to manufacturer's instructions.

**Functional study of the c.354+2T>G variant**

To study the effect of newly identified *MIA3* variant (c.354+2T>G) on splicing, total RNA was extracted from the patient’s leukocytes using QIAamp RNA Blood Mini Kit (Qiagen, Germany). Five µg of total RNA were reverse transcribed into cDNA using QuantiTect Reverse Transcription Kit (Qiagen, Germany). The synthesized cDNA was then used as a template for partial amplification of the *MIA3* gene (from exons 2 to 4) using one pair of primers: 5’- GCGGACGACGAATGCAG -3’ and 5’- TGGCACTTTTAGTTTATCTTGCAG -3’ under the following conditions: 96°C for 5 min, a total of 30 cycles of 96°C for 45 sec, annealing at 62°C for 45 sec, 72°C for 45 sec, and a final extension of 72°C for 5 min. PCR products were separated by 2% agarose gel electrophoresis and then purified and sequenced as described above.
