# Supplementary figures and images for: Deciphering the phenotypic spectrum associated with MIA3-related odontochondrodysplasia
Source: J Hum Genet. 2025 Mar 21;70(5):257–63. doi: 10.1038/s10038-025-01328-y (PMC11964919; doi:10.1038/s10038-025-01328-y)

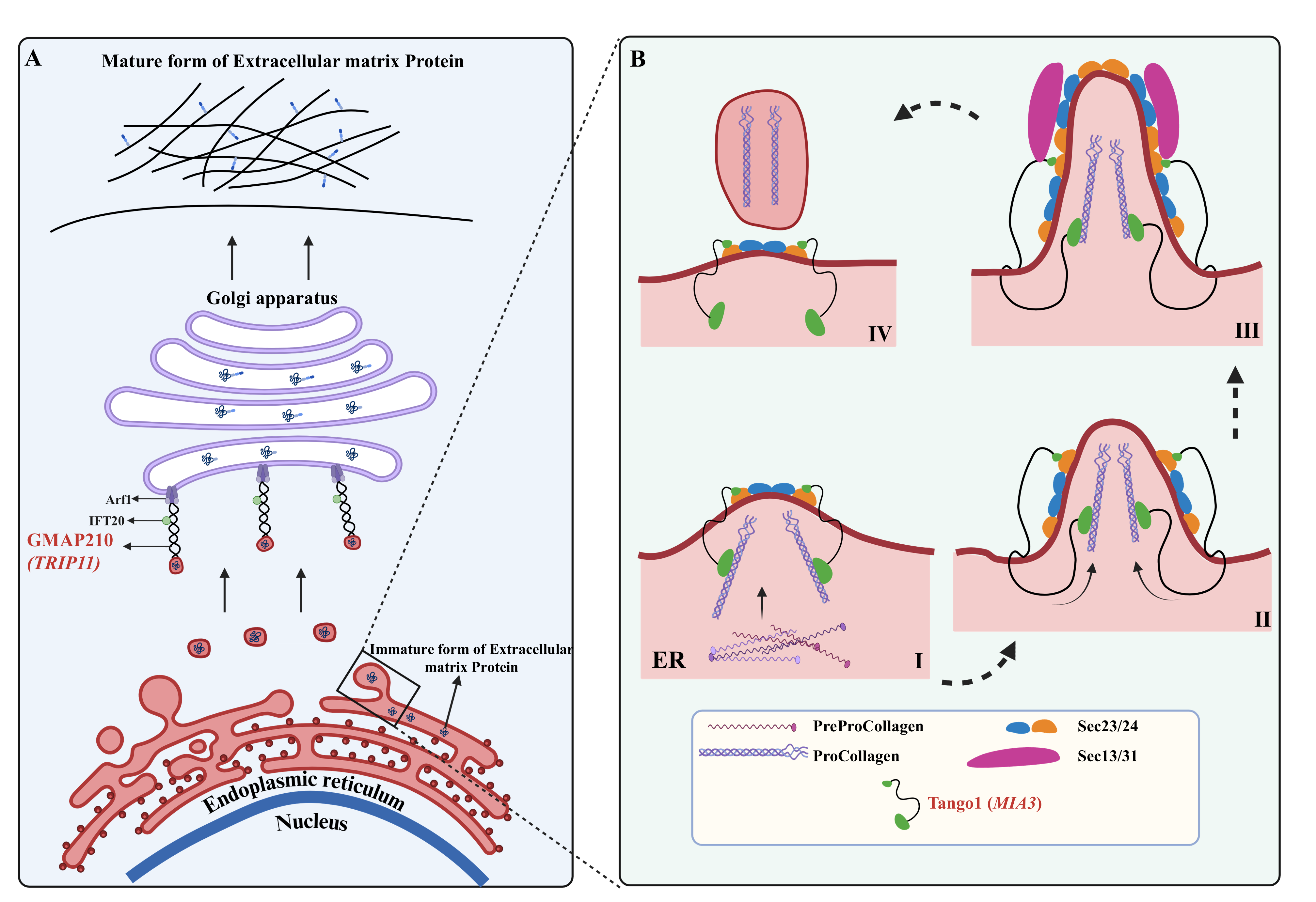

Supplement: Supplementary file 2 — Supplementary Figure 1 [file 10038_2025_1328_MOESM2_ESM.tif]
