# Supplementary material for: Deciphering the phenotypic spectrum associated with MIA3-related odontochondrodysplasia
Source: J Hum Genet. 2025 Mar 21;70(5):257–63. doi: 10.1038/s10038-025-01328-y (PMC11964919; doi:10.1038/s10038-025-01328-y)
Supplement: Supplementary file 3 — Supplementary Table 1 [file 10038_2025_1328_MOESM3_ESM.docx]

**Supplementary Table 1:** In silico prediction scores, population frequency, and conservation score of the identified *MIA3* variants

|  | ***MIA3***  (NM_198551) (nucleotide change, amino acid change, exon) | |
| --- | --- | --- |
|  | **c.113G>T**  **p.Cys38Phe**  **Exon: 1** | **c.354+2T>G**  **p.Val90_Asp118del**  **Exon3/ Intron 3** |
| **SIFT** | Deleterious (0) | - |
| **SIFT4G** | Pathogenic Supporting (0.008( |  |
| **REVEL** | Uncertain (0.652) | - |
| **CADD** | 24.2 | 33 |
| **MetaRNN** | Pathogenic Moderate (0.8619) | - |
| **BayesDel addAF** | Pathogenic Supporting (0.3687) | Pathogenic Moderate (0.3332) |
| **BayesDel noAF** | Pathogenic Supporting(0.2918) | Pathogenic Supporting (0.2408) |
| **Mutation Taster** | Disease causing (0.999) | Disease causing (1) |
| **AlphaMissense** | Pathogenic Moderate (0.9635) | - |
| **M-CAP** | Pathogenic Moderate (0.9878) |  |
| **MutPred** | Pathogenic Moderate (0.85) | - |
| **PROVEAN** | Pathogenic Moderate (-7.2) | - |
| **MetaRNN** | Pathogenic Moderate (0. 8619) | - |
| **MutPred** | Uncertain (0.543) | - |
| **dbscSNV** | - | Pathogenic Strong (0.9999) |
| **MaxEntScan** | - | Pathogenic Moderate (7.6472) |
| **EIGEN** | - | Pathogenic Moderate (1.0194) |
| **Splice AI**  **Doner gain delta score**  **Doner loss delta score** | - | 0.1  1.0 |
|  |  |  |
| **ACMG classification** | VUS  PM2, PP3 | Pathogenic  PVS1, PM2 PM4, PP3 |
| **gnomAD v.4 exome allele frequency** | 0 | 0 |
| **Conservation Scores phyloP100** | 4.4 | 7.271 |
