# Supplementary material for: Deciphering the phenotypic spectrum associated with MIA3-related odontochondrodysplasia
Source: J Hum Genet. 2025 Mar 21;70(5):257–63. doi: 10.1038/s10038-025-01328-y (PMC11964919; doi:10.1038/s10038-025-01328-y)
Supplement: Supplementary file 4 — Supplementary Figure 1 Legend [file 10038_2025_1328_MOESM4_ESM.docx]

**Supplementary Figure 1:** (A) GMAP210-mediated vesicle tethering to the Golgi apparatus. GMAP210 tethers vesicles that originate from the ER and contain newly synthesized extracellular matrix proteins, such as collagen, to the Golgi apparatus. It also plays a role in recycling Golgi enzymes that are critical for modifying these proteins. Proper modification in the Golgi is essential for the proteins’ function in the extracellular matrix. Pathogenic variants in in *TRIP11*, which encodes GMAP210, can impair these processes, contributing to developmental disorders like odontochondrodysplasia. (B) TANGO1-mediated collagen export from the ER. TANGO1 facilitates the export of procollagen from the ER by organizing and packaging these large molecules into vesicles. During vesicle formation (Stage I to IV), TANGO1 helps the vesicles grow large enough to accommodate procollagen. Once the vesicles are fully formed, they transport procollagen to the Golgi for further maturation. Disruption of TANGO1 (encoded by *MIA3*) can impair collagen trafficking, leading to skeletal abnormalities such as those seen in odontochondrodysplasia.
